# Biomechanics of anterior endocrowns with different designs and depths: Study of finite elements

**DOI:** 10.4317/jced.60889

**Published:** 2023-12-01

**Authors:** Fredy-Hugo Cruzado-Oliva, Luis-Felipe Alarco-La Rosa, Alexander Vega-Anticona, Heber-Isac Arbildo-Vega

**Affiliations:** 1 Escuela de Estomatología, Facultad de Estomatología, Universidad Nacional de Trujillo. Trujillo – Perú; 2 Escuela de Ingeniería de Materiales, Facultad de Ingeniería, Universidad Nacional de Trujillo. Trujillo – Perú

## Abstract

**Background:**

To date, there is no clear consensus in the literature on which endocrown design and depth is the most effective treatment option for restoring endodontically treated maxillary central incisors. Aim: To determine the stress distribution of the anterior endocrowns by means of finite element analysis.

**Material and Methods:**

Nine 3D finite element models (groups A – I) were made, each one representing a restoration system of endodontically treated upper central incisors. The models were endocrowns with and without ferrules at 0, 1, 3, and 5 mm depth and a post-core stump control group. A static load of 100N of force was applied to the palatal face at 45º from the long axis of the tooth. The Von Mise values and the maximum stress in the crown, dentin and resin cementum were evaluated separately.

**Results:**

The maximum stress distribution was C < B < A < D < H < F < E < G < I and the Von Mises stresses were in the upper 1/3 of the retainer of endocrowns A, B, C and D. ; in the vestibular neck in endocrowns E and F; in the final 1/3 of the retainer in the endocrown G; in the middle 1/3 of the retainer in the H endocrown; and at the level of the vestibular neck of the crown in model I.

**Conclusions:**

The smallest distribution of maximum and Von Mises stresses was observed in model C.

** Key words:**Finite element analysis, Biomechanics, upper central incisor, endodontically treated teeth, dental restoration.

## Introduction

The upper central incisors are the teeth that present the highest frequency of traumatic dental injuries, these coronary fractures involve pulpal exposures, where root canal treatment is necessary and later its restoration (1). Furthermore, these teeth are involved in the protection of the posterior teeth in protrusive movements. Likewise, they have the function of tearing food and the stresses that arise during tearing and movements are extremely important for the long-term success of post-endodontic restorations (2).

Endodontically treated teeth present quantitatively and qualitatively very different mechanical properties compared to vital teeth; For this reason, they are a common problem in restorative dentistry, commonly linked to fractures that occur in said teeth. mainly due to the volumetric loss of its structural integrity, given by the preparation of the endodontic access cavity, the instrumentation of the root canal, the obturation technique or the preparation and the inadequate selection of the post space (3,4). Therefore, the optimal way to restore teeth after endodontic treatment remains a controversial topic of heated debate to this day (5).

Treatments have been sought that range from direct aesthetic restorations with composite, conventional indirect restorations with posts, cores and prosthetic crowns, however, to restore the latter, it is necessary to prepare the root canal, as well as the supragingival tissues, unfortunately this carries the risk bacterial contamination and root perforation, as well as coronary wear (5,6).

Currently, with the advancement of adhesive techniques and an increasing emphasis on minimally invasive dentistry, endocrowns have been proposed as an excellent alternative for endodontically treated teeth (7-9). Due to the fact that this restoration is simpler and simpler (10), where the crown and the nucleus are united, there is less clinical time, less dental wear; Also, no post is needed; therefore, it reduces the probability of root fracture (5).

Endocrowns have a high survival rate of 91.4% at 5 years, but reported only in posterior teeth (11), however, there is little and controversial scientific evidence in anterior teeth. The success of endocrowns depends on the micromechanical retention provided by the design, that is, the existing ferrule effect or not, and the depth of its extension in the pulp chamber, these play a fundamental role in the distribution of tensions of restorative therapies of endodontically treated teeth. Therefore, the objective of our study was to determine the biomechanical behavior of anterior endocrowns with different designs and depths using finite element analysis.

## Material and Methods

-Study design:

This was an experimental, cross-sectional, comparative and *in vitro* study and was carried out using finite element analysis, where the study variables were anterior endocrown restorations with different preparation design (with and without ferrule) and depth of extension in the root canal (0mm, 1mm, 3mm and 5mm), in addition a control group was made with crown stump restoration. This research was approved by the Ethics Committee of the Faculty of Stomatology of the National University of Trujillo, P.I.B. ITS T. – 010 - 2023.

**Figure 1:**
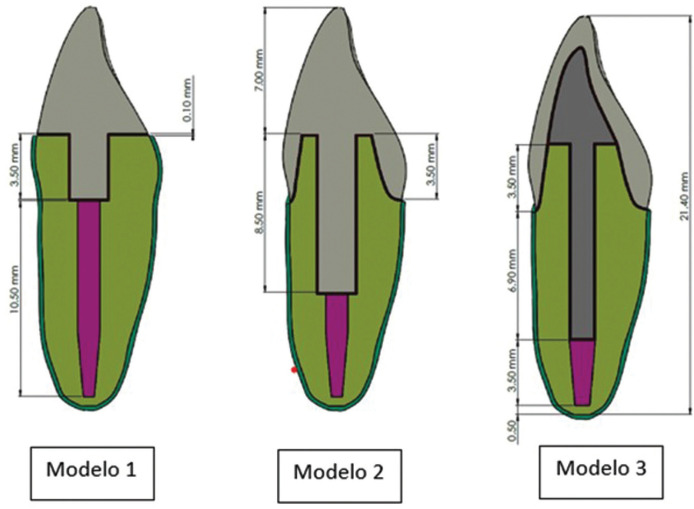
Dimensions of the models.

-Preparation of solid models and finite elements:

A maxillary central incisor was collected, with a crown length of 10.5 mm and a medial-distal width of 8.5 mm, and with a root of 11 mm, recently extracted due to periodontal disease, which all its surfaces were scanned with a Multifunctional 3D Dental scanner UP560 (3DBiotech, China), said scan in STL format was processed with the Meshmixer software, in which it was smoothed and reduced the number of elements to make the tooth more uniform, then it was saved in SLDDRW format , to be able to operate with the SOLIDWORKS software. In this last software, we proceeded to identify the X (mesial), Y (longitudinal of the tooth) and Z (vestibular) planes of the tooth and to make the cuts in the planes in such a way that a solid dental piece is obtained. Subsequently, three restoration models were made, one with an endocrown without a ferrule (Model 1) and with a ferrule (Model 2) varying their depths, and another as a control group with post and stump made to measure (Model 3) (Fig. 1).

For model 1, the maxillary central incisor was sectioned in a plane perpendicular to the longitudinal axis of the tooth at a distance of 7 mm from the incisal edge. Then, an 8 mm long by 2 mm wide cylinder was created to be inserted into the pulp chamber and root canal of the tooth. The sectioned crown and cylinder volumes were merged and added to the tooth models at different depths. Finally, a 0.1 mm thick layer of cement was cast around the entire endocrown.

For model 2, the same as design 1 were made, adding the ferrule effect, cutting the remaining coronary surface of the tooth as for a prosthetic crown abutment, with an inclination of the axial walls of 10º and a rounded shoulder termination line (Chamfer) of 0.8mm.

For model 3, the same was done for design 1 and 2, adding a fiberglass post (3M) and stump, for this, a cylinder 12 mm long by 1.2 mm wide was created, this solid it was inserted and added into the pulp chamber and root canal of the tooth model. In this way, a 1.2 mm diameter, 6.9 mm long post tooth was created, leaving 3.5 mm of gutta-percha at the root apex.

Subsequently, the designs presented were entered into the finite element method program of the ANSYS software for the respective simulation. The nodes and elements of each model are shown in Table 1.

**Table 1:**
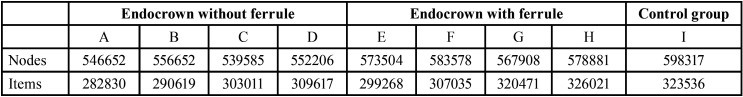
Number of nodes and elements of the different models analyzed with the finite element method.

-Design of models and groups:

A model was made with a 3.5mm high dental remnant. Nine groups of models with different designs and restoration depths were established.

A. Group of endocrowns without ferrule at 0 mm depth.

B. Group of endocrowns without ferrule at 1 mm depth.

C. Group of endocrowns without ferrule at 3 mm depth.

D. Group of endocrowns without ferrule at 5 mm depth.

E. Group of endocrowns with ferrule at 0 mm depth.

F. Group of endocrowns with ferrule at 1 mm depth.

G. Group of endocrowns with ferrule at 3 mm depth.

H. Group of endocrowns with ferrule at 5 mm depth.

I. Group with fiber stump and crown.

-Experimental assumptions, boundary conditions and parameter settings:

Where it is assumed that all the materials and tissues in the model are linear elastomers with continuous homogeneous isotropy and that they met small strain conditions, the specific mechanical parameters of each material are (12,13): (Table 2).

**Table 2:**
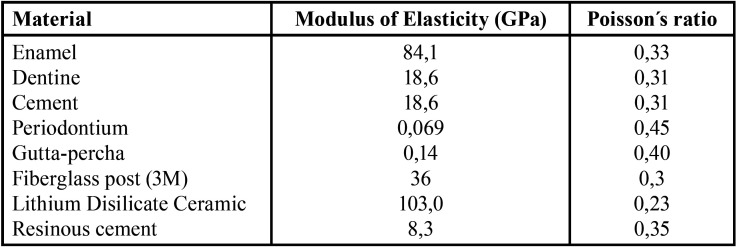
Mechanical parameters of the materials.

The endocrowns and the crown were manufactured based on Lithium Disilicate ceramic as they are considered the best restorative materials due to their properties of greater mechanical resistance, the strong adhesion force to the tooth structure and the superior aesthetic appearance (14). Likewise, Variolink II resinous cement (Ivoclar, Vivadent AG, Schaan, Lichtenstein) was used, since it is a cement with a fourth-generation adhesive system, which offers better adhesion strength (6).

-Charging methods:

A clinical occlusal load of 100N of static load force was simulated in a direction of 45º with the long axis of the tooth, in the middle 1/3 of its palatal surface and with a load area of 2 mm2. Since it is known that the maximum force of the average upper central incisor is 12 Kg (117.2N) (2).

-Voltage charging methods and indicators.

The distribution and maximum Von Mises stresses were analyzed in the different components involved in the restoration (crown, dentin, and resinous cement).

## Results

**Figure 2:**
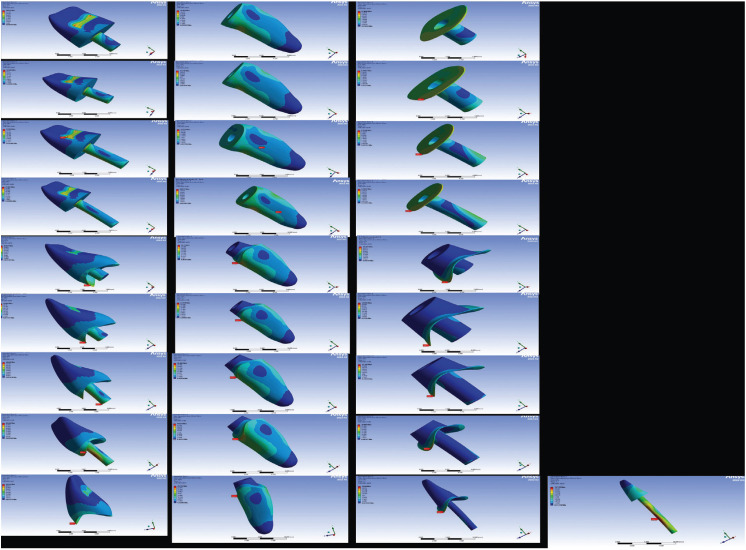
Von Mises stress distribution.

The Von Mises stress distribution of the crown, dentin, and resin cementum is shown in Fig. 2. Where it can be seen that the Von Mises stress distribution in the crown was: in groups A, B, C and D in the upper 1/3 of the endocrown retainer; groups E and F, in the vestibular neck of the endocrown; group G, in the final 1/3 of the endocrown retainer; group H, in the middle 1/3 of the endocrown retainer; and group I, in the vestibular neck of the crown. The distribution of Von Mises stresses in the dentin was: in groups A, B, E, F, G, H and I it was in the upper buccal 1/3 of the remaining dental tissue and in groups C and D, in the 1/3 half labial. And the distribution of Von Mises stresses in the cement was: in groups A, B, E, F, G, H and I it was similar and distributed throughout the adhesive layer, and in groups C and D, it is distributed by the entire adhesive layer and lower 1/3 of the endocrown retainer in palatal view. In addition, in group I, the stress distribution area in the post was observed at the level of its middle 1/3.

In relation to the Von Mises stress distribution of the crown, dentin, and resin cement, it was found that the stress distribution of endocrowns in dentin and cement is approximately half of that experienced in the crown. Anterior endocrowns with ferrules present similar biomechanical behavior as fiberglass post-die crowns, in addition they present a greater stress distribution than endocrowns without ferrules. Endocrowns without Ferrule present approximately 1.7, 2.4, and 5.1 times lower stress distribution than endocrowns with Ferrule in the crown, dentin, and cementum, respectively. And the endocrowns present a lower stress distribution in groups C and D, both in the crown, dentin and cement (Table 3).

**Table 3:**
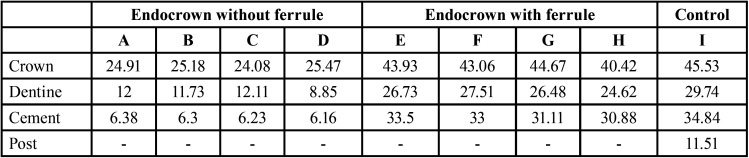
Values of the maximum Von Mises stresses in the restored teeth in MPa.

## Discussion

Due to limited information, what design and depth of endocrown restorations of anterior teeth is most beneficial. The few studies involving this type of restoration are based on static fracture resistance and variables cannot be manipulated (15–21). In the present study, a three-dimensional finite element analysis was carried out, which allows us to quantify and characterize the stress distribution after having applied the external functional forces (22).

The distribution of maximum stresses in the anterior endocrowns is reflected from highest to lowest in the crown, dentin and cement, due to the proximity of the incidence of force on the structures involved (2,6).

The lowest stress distribution was found in endocrowns without ferrules, both in the crown, dentin and cementum, compared to endocrowns with ferrules; because the non-ferruled endocrowns were prepared with a butt union, which led to maintaining a peripheral enamel band, while the ferruled preparation was prepared with a shoulder finish line, which caused loss of healthy tissue of enamel and dentin that would be important for the adequate support and adhesion of the restoration (2,23,24), therefore, in the endocrowns without ferrules, more dental tissue was preserved, which was more propitious for the uniform distribution of the stresses in the tooth (2,25). However, in one study it was found that the greatest resistance to fracture occurs in endocrowns with ferrules (16).

The distribution of stresses of the endocronals according to their depth varied, the greater the depth, the greater the stress, similar data found in other studies (2,15,18), which may be due to an increase in the contact surface area (17). However, in one study it was found that teeth restored with endocrowns and fiberglass-reinforced posts have similar fracture strength, because endocrowns behave like short posts (19). Contrary to other studies, which conclude that the depth of the endocrown preparation does not have significant consequences on the resistance to fracture (15), nor on the distribution of stresses (2).

When comparing the design and depth in our study, it was observed that group C presents a lower stress distribution in the crown, results similar to those of Li *et al*. (2). And in dentin and cement, group D is slightly less than group C. This is corroborated by the analysis carried out with the Von Misses Failure theory, where the highest color intensity for both groups was similar, in 1/3 upper crown, in the labial middle 1/3 of the dentin and in the cementum, at the vestibular level and in the lower palatal 1/3. Due to the similar characteristics, a greater extension in the root depth is not necessary, which would lead to a greater preparation of the root canal and, therefore, a greater weakness of the teeth treated with endodontics (20).

In our study, the stress distribution of endocrowns without ferrules were much lower than traditional restorations with crown stump posts, results that coincide with other studies (21,26,27), This is due to the fact that the fewer the number of interfaces between different materials, it will support greater tensions, as observed in the monolithic nature of the endocrowns (2,15), therefore, it reduces the cement layer; Contrary to what happens with crown posts, which need three or four layers of composite resin to customize them, otherwise the cement layer is very thick; therefore, prone to fracture (28). Furthermore, if the endocrown tooth is to fracture, the location of the fracture would be in the neck of the tooth (6) and the affected tooth could be repaired again by crown lengthening surgery or orthodontic traction, compared to post-core crown core (2,29).

On the other hand, in our study a similar biomechanical behavior was observed between endocrowns with ferrules and post-die crowns, probably due to the substantial loss of tooth structure when preparing, which leads to less adhesive bonding surface, in addition, cementation is only on dentin substrate. Similar results to Hofsteenge JW and Gresnigt M (20).

Due to the rapid mechanical analysis and thanks to its versatility, the finite element method is currently widely used; but the complex forces that are generated in the oral cavity cannot yet be simulated. For this reason, one of the limitations of this study is that it only analyzes ideal stress conditions, that is, static loads, contrary to what happens in real chewing, where the forces are dynamic. However, with the advancement of finite element analysis technology, it could be used to study biomechanics in more detail. The authors recommend that the results of this study should be confirmed by clinical trials and by further *in vitro* mechanistic testing.

## Conclusions

The presence of dental remnants in anterior tooth restorations is extremely important for stress distribution; however, it is not necessary to perform the ferrule effect. Endocrowns without ferrules present lower Von Mises stresses, therefore, they have greater mechanical resistance. Considering the effect of the depth of the endocrown without ferrule and comparing the maximum stresses obtained, group C would be the most optimal.

## References

[1] Donnelly A, Foschi F, McCabe P, Duncan HF (2022). Pulpotomy for treatment of complicated crown fractures in permanent teeth: A systematic review. Int Endod J.

[2] Li X, Kang T, Zhan D, Xie J, Guo L (2020). Biomechanical behavior of endocrowns vs fiber post-core-crown vs cast post-core-crown for the restoration of maxillary central incisors with 1 mm and 2 mm ferrule height: A 3D static linear finite element analysis. Medicine (Baltimore).

[3] Hayes A, Duvall N, Wajdowicz M, Roberts H (2017). Effect of Endocrown Pulp Chamber Extension Depth on Molar Fracture Resistance. Oper Dent.

[4] Kassis C, Khoury P, Mehanna CZ, Baba NZ, Bou Chebel F, Daou M (2021). Effect of Inlays, Onlays and Endocrown Cavity Design Preparation on Fracture Resistance and Fracture Mode of Endodontically Treated Teeth: An In Vitro Study. J Prosthodont.

[5] Carvalho MA de, Lazari PC, Gresnigt M, Del Bel Cury AA, Magne P (2018). Current options concerning the endodontically-treated teeth restoration with the adhesive approach. Braz Oral Res.

[6] Dejak B, Młotkowski A (2018). Strength comparison of anterior teeth restored with ceramic endocrowns vs custom-made post and cores. J Prosthodont Res.

[7] Govare N, Contrepois M (2020). Endocrowns: A systematic review. J Prosthet Dent.

[8] Soliman M, Alshamrani L, Yahya B, Alajlan G, Aldegheishem A, Eldwakhly E (2021). Monolithic Endocrown Vs. Hybrid Intraradicular Post/Core/Crown Restorations for Endodontically Treated Teeth; Cross-sectional Study. Saudi J Biol Sci.

[9] Deulkar PV, Bane SP, Rathi NV, Thosar NR (2022). Rehabilitation of Traumatised Maxillary Anterior Teeth in Children Using Endocrown: A Case Series. Cureus.

[10] Papalexopoulos D, Samartzi TK, Sarafianou A (2021). A Thorough Analysis of the Endocrown Restoration: A Literature Review. J Contemp Dent Pract.

[11] Al-Dabbagh RA (2021). Survival and success of endocrowns: A systematic review and meta-analysis. J Prosthet Dent.

[12] Zheng Z, Sun J, Jiang L, Wu Y, He J, Ruan W (2022). Influence of margin design and restorative material on the stress distribution of endocrowns: a 3D finite element analysis. BMC Oral Health.

[13] Meng Q, Zhang Y, Chi D, Gong Q, Tong Z (2021). Resistance fracture of minimally prepared endocrowns made by three types of restorative materials: a 3D finite element analysis. J Mater Sci Mater Med.

[14] Haralur SB, Alamrey AA, Alshehri SA, Alzahrani DS, Alfarsi M (2020). Effect of different preparation designs and all ceramic materials on fracture strength of molar endocrowns. Journal of Applied Biomaterials & Functional Materials.

[15] Kanat-Ertürk B, Saridağ S, Köseler E, Helvacioğlu-Yiğit D, Avcu E, Yildiran-Avcu Y (2018). Fracture strengths of endocrown restorations fabricated with different preparation depths and CAD/CAM materials. Dent Mater J.

[16] Silva-Sousa AC, Moris ICM, Barbosa AFS, Silva-Sousa YTC, Sousa-Neto MD, Pires CRF (2020). Effect of restorative treatment with endocrown and ferrule on the mechanical behavior of anterior endodontically treated teeth: An in vitro analysis. J Mech Behav Biomed Mater.

[17] Bozkurt DA, Buyukerkmen EB, Terlemez A (2023). Comparison of the pull-out bond strength of endodontically treated anterior teeth with monolithic zirconia endocrown and post-and-core crown restorations. J Oral Sci.

[18] Einhorn M, DuVall N, Wajdowicz M, Brewster J, Roberts H (2019). Preparation Ferrule Design Effect on Endocrown Failure Resistance. Journal of Prosthodontics.

[19] Ramírez-Sebastià A, Bortolotto T, Cattani-Lorente M, Giner L, Roig M, Krejci I (2014). Adhesive restoration of anterior endodontically treated teeth: influence of post length on fracture strength. Clin Oral Invest.

[20] Hofsteenge JW, Gresnigt M (2021). The Influence of Dentin Wall Thickness and Adhesive Surface in Post and Core Crown and Endocrown Restorations on Central and Lateral Incisors. Oper Dent.

[21] de Carvalho MA, Lazari-Carvalho PC, Del Bel Cury AA, Magne P (2021). Accelerated fatigue resistance of endodontically treated incisors without ferrule restored with CAD/CAM endocrowns. Int J Esthet Dent.

[22] Rodrigues YL, Mathew MT, Mercuri LG, da Silva JSP, Henriques B, Souza JCM (2018). Biomechanical simulation of temporomandibular joint replacement (TMJR) devices: a scoping review of the finite element method. Int J Oral Maxillofac Surg.

[23] Sedrez-Porto JA, Rosa WL de O da, da Silva AF, Münchow EA, Pereira-Cenci T (2016). Endocrown restorations: A systematic review and meta-analysis. Journal of Dentistry.

[24] Elagra DME (2019). Endocrown preparation: Review. International Journal of Applied Dental Sciences.

[25] Guo J, Wang XY, Li XS, Sun HY, Liu L, Li HB (2016). [Influence of different designs of marginal preparation on stress distribution in the mandibular premolar restored with endocrown]. Nan Fang Yi Ke Da Xue Xue Bao.

[26] El-Damanhoury HM, Haj-Ali RN, Platt JA (2015). Fracture resistance and microleakage of endocrowns utilizing three CAD-CAM blocks. Oper Dent.

[27] Bankoğlu Güngör M, Turhan Bal B, Yilmaz H, Aydin C, Karakoca Nemli S (2017). Fracture strength of CAD/CAM fabricated lithium disilicate and resin nano ceramic restorations used for endodontically treated teeth. Dent Mater J.

[28] Fernandes V, Silva AS, Carvalho O, Henriques B, Silva FS, Özcan M (2021). The resin-matrix cement layer thickness resultant from the intracanal fitting of teeth root canal posts: an integrative review. Clin Oral Investig.

[29] Zhen M, Wei YP, Hu WJ, Rong QG, Zhang H (2016). [Finite element analysis of the maxillary central incisor with traditional and modified crown lengthening surgery and post-core restoration in management of crown-root fracture]. Zhonghua Kou Qiang Yi Xue Za Zhi.

